# Bio‐Derived Hard Carbon from *Bassia scoparia* as a Multifunctional Reinforcement for Chlorinated Polypropylene: Structural, Thermal, and Bioactive Performance

**DOI:** 10.1002/open.70241

**Published:** 2026-06-01

**Authors:** Gokhan Acik, Esra Kulaksiz Alayont, Burcu Acik, Gulcan Kuyucuklu Kazan, Derya Altintas, Hakan Kolancilar, Yuksel Bayrak

**Affiliations:** ^1^ Arda Vocational School Department of Chemistry and Chemical Processing Technologies Trakya University Edirne Türkiye; ^2^ Faculty of Science Department of Chemistry Trakya University Edirne Türkiye; ^3^ Institute of Science Department of Chemistry Trakya University Edirne Türkiye; ^4^ Faculty of Medicine Department of Medical Microbiology Kırklareli University Kırklareli Türkiye; ^5^ Arda Vocational School Department of Pharmacy Services Trakya University Edirne Türkiye; ^6^ Faculty of Pharmacy Department of Professional Pharmaceutical Sciences Trakya University Edirne Türkiye

**Keywords:** antibacterial activity, antioxidant activity, *Bassia scoparia*, chlorinated polypropylene, hard carbon, multifunctional reinforcement

## Abstract

The development of sustainable multifunctional polymer composites with enhanced thermal, mechanical, surface, and bioactive properties is important for advanced coating and packaging applications because conventional polypropylene (PP)‐based materials generally lack intrinsic antioxidant and antibacterial functionality. In this study, nitric acid‐treated hard carbon derived from *Bassia scoparia* biomass (N‐BSHC) was utilized as a sustainable multifunctional reinforcement for chlorinated polypropylene (PP‐Cl) composite films. Composite films containing 1.0, 1.5, and 2.5 wt% N‐BSHC were fabricated by solution casting and characterized using Fourier transform infrared, scanning electron microscope‐energy‐dispersive X‐ray (SEM‐EDX), water contact angle (WCA), thermogravimetric analysis, differential scanning calorimetry, mechanical testing, and antioxidant and antibacterial assays. SEM‐EDX analysis confirmed homogeneous filler distribution and the presence of oxygen‐ and nitrogen‐containing surface functionalities, which improved matrix compatibility and surface wettability. The WCA decreased from 105° for neat PP‐Cl to 94° for the composite containing 2.5 wt% filler. Thermal degradation temperatures increased from 338°C/421°C to 346°C/428°C, while the elastic modulus increased from 48.56 to 73.61 MPa at 1.5 wt% filler loading. Furthermore, the composites exhibited antioxidant activity and strong antibacterial performance against *Staphylococcus aureus* and *Escherichia coli*. These findings demonstrate that biomass‐derived N‐BSHC is an effective sustainable filler for advanced PP‐Cl composites.

## Graphical abstract

Nitric acid‐functionalized biomass‐derived hard carbon was successfully incorporated into chlorinated polypropylene films. Enhanced thermal stability, wettability, antioxidant activity, and antibacterial performance were achieved through improved matrix–filler interactions and homogeneous filler dispersion, demonstrating the potential of the developed multifunctional composite films for advanced coating and packaging applications.

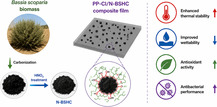

## 1 Introduction

The increasing demand for multifunctional polymer composites with enhanced surface, thermal, and bioactive properties has driven significant interest in the incorporation of bio‐derived and chemically modified fillers into commodity polymers [1]. Polypropylene (PP) is one of the most widely used thermoplastic polymers due to its low density, chemical resistance, mechanical robustness, and cost‐effectiveness [2]. Due to these superior features, it is a cornerstone material in high‐volume industrial sectors such as packaging, automotive components, coatings, and consumer goods [3]. However, its nonpolar chemical structure and low surface energy severely limit interfacial adhesion, surface wettability, and functional integration with polar or bio‐derived fillers [4]. These inherent drawbacks restrict the use of neat PP in advanced applications requiring enhanced surface activity, coating adhesion, or bioactive functionality. As a result, modified PP systems, particularly chlorinated polypropylene (PP‐Cl), have attracted increasing attention as alternative matrices for multifunctional composite materials.

PP‐Cl is obtained by partial chlorination of the PP backbone, introducing polar C—Cl bonds that significantly increase surface energy, polarity, and chemical reactivity while preserving the advantageous bulk properties of PP [5, 6]. This structural modification improves compatibility with polar fillers and coatings, enhances interfacial adhesion, and facilitates uniform dispersion of functional additives. Consequently, PP‐Cl is widely employed in applications such as adhesion promoters, functional coatings, and compatibilizers, where neat PP fails to deliver adequate performance [7]. Nevertheless, PP‐Cl itself remains largely inert in terms of antioxidant and antibacterial activity, necessitating further functionalization strategies.

In parallel, bio‐derived hard carbon (HC) materials have gained increasing attention as sustainable and multifunctional fillers for polymer composites due to their high thermal stability, tunable surface chemistry, large specific surface area, and inherent carbon‐rich structure [8, 9]. HC obtained from biomass sources offers a viable route for valorizing renewable resources while enabling improvements in mechanical reinforcement, thermal resistance, and surface‐related properties of polymer matrices [10, 11]. Moreover, depending on the precursor and post‐treatment strategy, HC can contribute additional functionalities such as antioxidant and antibacterial activity, making it particularly attractive for advanced composite films and coating applications [12, 13, 14].

Among various biomass resources, *Bassia scoparia*, an abundant and fast‐growing plant species [15], represents a promising yet underutilized precursor for HC production. In this study, *B. scoparia* was first converted into HC, which was subsequently treated with nitric acid to tailor its surface chemistry. This postcarbonization nitric acid treatment introduces oxygen‐ and nitrogen‐containing functional groups onto the HC surface, enhancing polarity, surface reactivity, and interfacial compatibility with polymer matrices [16, 17, 18]. Such surface‐functionalized HC is especially well suited for PP‐Cl systems, where improved matrix–filler interactions can be leveraged to achieve synergistic enhancements in surface wettability, thermal behavior, and bioactive performance.

In this work, nitric acid‐treated HC of *B. scoparia* (N‐BSHC) was incorporated into PP‐Cl at 1.0, 1.5, and 2.5 wt%, and composite films were fabricated via the solution casting method. The influence of filler content on the structural, morphological, surface, thermal, antioxidant, and antibacterial properties of the resulting composites was comprehensively evaluated using Fourier transform infrared (FT‐IR), scanning electron microscope‐energy‐dispersive X‐ray (SEM‐EDX), water contact angle (WCA) measurements, thermogravimetric analysis (TGA), differential scanning calorimetry (DSC), and in vitro antioxidant and antibacterial assays. Comparative analyses with neat PP‐Cl were conducted to elucidate the multifunctional role of N‐BSHC and to establish clear structure–property–function relationships.

From a sustainability perspective, the developed composite system offers potential environmental advantages due to the utilization of biomass‐derived HC obtained from *B. scoparia*, an abundant and renewable plant resource. Compared with conventional petroleum‐derived or mineral‐based reinforcing fillers, the conversion of biomass into functional carbon materials may contribute to reduced dependence on nonrenewable resources and improved waste valorization. In addition, the use of a metal‐free carbonaceous reinforcement eliminates concerns associated with metal‐containing antimicrobial additives. Although a quantitative life‐cycle assessment (LCA) or direct carbon footprint analysis was beyond the scope of the present work, the proposed approach provides a promising platform for the development of more sustainable multifunctional polymer composites. Future studies should focus on detailed environmental impact assessments, including energy consumption, carbon emissions, and end‐of‐life considerations associated with large‐scale production of biomass‐derived HC composites.

## 2 Experimental Part

### 2.1 Materials

PP‐Cl (chlorine content ~30 wt%) was used as the polymer matrix and supplied by a commercial manufacturer in pellet form. Analytical‐grade nitric acid (HNO_3_, 65%–68%), ethanol, and other reagents employed during the HC preparation and surface modification steps were purchased from Sigma–Aldrich and used as received without further purification. Tween 80 (polyoxyethylene sorbitan monooleate, for synthesis) was employed as a dispersing agent during composite formulation. *B. scoparia* biomass was collected locally from Lüleburgaz, Kırklareli (41° 24’ 24″ N/27° 21’ 19″ E) during June 2024, thoroughly washed with deionized water to remove surface impurities, and air‐dried prior to carbonization. Deionized water was used throughout all experimental procedures. For biological evaluations, 2,2‐diphenyl‐1‐picrylhydrazyl (DPPH) and 2,2′‐azinobis(3‐ethylbenzothiazoline‐6‐sulfonic acid) (ABTS) reagents were used for antioxidant activity assays, while ascorbic acid served as the reference antioxidant. Bacterial strains *Staphylococcus aureus ATCC 29213* and *Escherichia coli ATCC 25922* were obtained from certified culture collections and used for in vitro antibacterial testing. Standard antibiotics, including ampicillin, vancomycin, cefotaxime, ciprofloxacin, gentamicin, and imipenem, were used as controls in antimicrobial susceptibility assays. All microbiological media and consumables were of microbiological grade and prepared according to the manufacturers’ instructions.

### 2.2 Preparation of Hard Carbon of *B. scoparia* and its Nitric Acid‐Treated Derivative

Preliminary optimization experiments involving different nitric acid concentrations and treatment durations were conducted prior to the final composite preparation. Lower oxidation severity resulted in insufficient surface modification, whereas more aggressive treatment conditions caused partial structural deterioration and increased aggregation tendency of the carbon material. Therefore, the selected treatment conditions were considered suitable for achieving effective surface functionalization while preserving the integrity of the HC framework. The nitric acid treatment is proposed to oxidatively activate defect sites and edge carbon atoms on the HC surface, promoting the formation of oxygen‐containing functional groups such as hydroxyl, carbonyl, and carboxyl moieties. In addition, limited nitrogen‐containing surface functionalities may be introduced during the oxidation process. These surface polar groups improve the hydrophilicity and interfacial compatibility of the HC with the PP‐Cl matrix. In this perspective, dried *B. scoparia* biomass was first mechanically ground and sieved to obtain a homogeneous precursor powder. The powder was then subjected to carbonization in a tubular furnace under an inert nitrogen atmosphere. Dried B. scoparia biomass was carbonized at 800°C for 2 h under a continuous nitrogen atmosphere using a heating rate of 10°C min^−1^. The carbonization conditions were selected according to previous literature reports on biomass‐derived HC production to obtain a stable carbonaceous structure suitable for subsequent surface functionalization [12]. After cooling naturally to room temperature under nitrogen, the resulting carbonized product was collected and gently ground to obtain pristine HC derived from *B. scoparia* (BSHC). To tailor the surface chemistry and enhance the polarity of the HC, a postcarbonization nitric acid treatment was applied. Briefly, BSHC (1 g) was dispersed in a mixture of concentrated nitric acid and distilled water (350 mL, 3:4 (*v*/*v*)) under continuous stirring and maintained at 90°C for 3.5 h. This oxidative treatment promotes the formation of oxygen‐ and nitrogen‐containing functional groups on the carbon surface while preserving the carbonaceous framework. Following the treatment, the suspension was repeatedly washed with deionized water until a neutral pH was reached, ensuring complete removal of residual acid and soluble byproducts. The solid was then filtered and dried in a vacuum oven to obtain nitric acid‐treated hard carbon (N‐BSHC). The resulting N‐BSHC powder was lightly ground prior to composite preparation to minimize agglomeration and to facilitate uniform dispersion within the polymer matrix.

### 2.3 Sample Formulation

Neat PP‐Cl and PP‐Cl/N‐BSHC composite samples were prepared by a solution‐based formulation approach. PP‐Cl was first dissolved in THF under magnetic stirring at room temperature until a clear and homogeneous polymer solution was obtained. Then predetermined amounts of N‐BSHC were dispersed in the prepared solution using Tween 80 (two droplets by Pasteur pipette) as a nonionic dispersing agent to promote uniform particle distribution and to suppress agglomeration. After continuous stirring for 15 min, followed by additional sonication (5 min), the composite formulations were casted onto Petri dishes and dried at 35°C for 48 h to ensure effective evaporation of THF prior to characterization. Owing to the high volatility of THF, no visible solvent residue, tackiness, or deformation was observed in the obtained freestanding films after drying. Solution casting was selected as the fabrication method because it enables effective dispersion of nitric acid‐treated HC within the PP‐Cl matrix under mild processing conditions. In addition, the method facilitates the preparation of uniform thin films with improved matrix–filler interfacial interaction while minimizing possible filler agglomeration and thermal degradation that may occur during high‐temperature melt‐processing techniques. The good solubility of PP‐Cl in THF also makes solution casting a practical and efficient approach for producing homogeneous composite films. The formulations containing 1.0, 1.5, and 2.5 wt% N‐BSHC with respect to the polymer content were denoted as PP‐Cl/N‐BSHC‐1.0, PP‐Cl/N‐BSHC‐1.5, and PP‐Cl/N‐BSHC‐2.5, respectively. These concentrations were selected based on preliminary dispersion observations and previous studies on carbon‐filled polymer composites, where low‐to‐moderate filler contents generally provide improved dispersion stability and interfacial interaction while minimizing severe particle agglomeration. For comparison, a neat PP‐Cl solution was prepared following the same procedure without the addition of N‐BSHC. The prepared solutions were used directly in the subsequent coating and film formation processes without further modification. The obtained films were freestanding and could be removed from the casting surface after solvent evaporation without noticeable mechanical damage. Although film thickness was not directly measured, all samples were prepared under identical solution casting conditions using the same casting volume and substrate dimensions to ensure comparable film geometry throughout the study.

### 2.4 Coating Procedure and WCA Analysis of the Samples

Coatings were prepared using a dip‐coating approach on standard glass microscope slides. Prior to coating, the slides were thoroughly cleaned by sequential washing with ethanol and deionized water, followed by drying at room temperature to remove surface contaminants. The cleaned slides were vertically immersed into the prepared neat PP‐Cl and PP‐Cl/N‐BSHC solutions. The dip‐coating process was conducted using a fixed immersion time and a constant withdrawal rate (82 mm min^−1^) to promote uniform coating deposition on the glass substrates. The coated substrates were then dried under ambient conditions to allow solvent evaporation and film formation. The surface wettability of the coated films was evaluated by static WCA measurements using the sessile drop method. A 5 µL of deionized water was gently deposited onto the film surface, and the contact angle was recorded after stabilization. For each sample, water contact angle measurements were performed at five different surface locations, and the reported values represent the mean ± standard deviation.

### 2.5 In Vitro Antioxidant Activity

The in vitro antioxidant activities of N‐BSHC, neat PP‐Cl, and PP‐Cl/N‐BSHC composite films were evaluated using the DPPH and ABTS radical scavenging assays. These methods were selected due to their reliability in assessing the free radical neutralization capability of solid materials and polymer‐based films [19, 20]. The DPPH assay is based on the reduction of the stable nitrogen‐centered DPPH radical, which exhibits a characteristic violet coloration in solution. In the presence of antioxidant‐active species, the DPPH radical accepts electrons or hydrogen atoms from the sample, leading to a decrease in absorbance intensity that reflects the radical scavenging capability of the material. Briefly, for the DPPH assay, a freshly prepared DPPH solution was mixed with the test samples and incubated under dark conditions for predetermined time intervals. After incubation, the decrease in absorbance was measured using a UV–vis spectrophotometer at the characteristic wavelength of DPPH. Ascorbic acid was used as a positive control antioxidant under identical conditions. Similarly, the ABTS assay utilizes the radical cation derived from ABTS•^+^, which is generated through oxidation of ABTS prior to analysis. The generated blue‐green ABTS radical cation is subsequently neutralized by antioxidant‐active species, causing a reduction in absorbance proportional to the radical scavenging capability of the tested material. The ABTS radical cation was generated prior to analysis and subsequently reacted with the test samples. After incubation, the absorbance reduction of the ABTS•^+^ solution was recorded spectrophotometrically. Measurements for both assays were conducted at 30 and 60 min to evaluate time‐dependent radical scavenging behavior. All antioxidant measurements were performed in triplicate, and the results are presented as mean ± standard deviation. The radical scavenging activity was calculated using Equation (1)

(1) $$\text{Radical scavencing activity}\left(\%\right)=\left(\frac{A_{0}-A_{s}}{A_{0}}\right)\times 100$$

where *A*_0_ and *A*_s_ represent the absorbance values of the control and the sample, respectively. All measurements were performed in triplicate, and the results are reported as mean values.

### 2.6 In Vitro Antibacterial Activity

The antibacterial performance of N‐BSHC and PP‐Cl/N‐BSHC composite films was investigated using both broth microdilution assays and a contact‐based antibacterial test adapted from ISO 22196. These complementary methods were selected to evaluate intrinsic antimicrobial potency as well as surface‐mediated antibacterial behavior. All antibacterial experiments were conducted in triplicate to ensure reproducibility of the obtained results.

#### 2.6.1 Antibacterial Susceptibility Testing by the Broth Microdilution Method

The antimicrobial susceptibility of N‐BSHC was evaluated using a broth microdilution method in accordance with the Clinical and Laboratory Standards Institute guidelines (M100) [21]. *S. aureus ATCC 29213* and *E. coli ATCC 25922* were used as representative Gram‐positive and Gram‐negative bacterial strains, respectively. These strains are widely accepted quality‐control organisms for antimicrobial susceptibility testing. Fresh bacterial cultures were obtained by incubating colonies on Mueller‐Hinton agar at 37°C for 18–24 h. The bacterial cells were suspended in sterile saline, and the turbidity was adjusted to match the 0.5 McFarland standards. The suspensions were subsequently diluted in cation‐adjusted Mueller‐Hinton broth to achieve a final inoculum concentration of ≈5 × 10^5^ CFU mL^−1^. Serial two‐fold dilutions of N‐BSHC were prepared in 96‐well microplates over a defined concentration range. Standard antibiotics, including ampicillin, vancomycin, cefotaxime, ciprofloxacin, gentamicin, and imipenem, were tested in parallel as reference controls. Each well received an equal volume of bacterial suspension, while sterility and growth controls were included to validate the assay. Following incubation at 37°C for 24 h, the minimum inhibitory concentration (MIC) was determined as the lowest concentration at which no visible bacterial growth was observed. All experiments were performed in triplicate to ensure reproducibility.

#### 2.6.2 In Vitro Antibacterial Activity Assessment Based on ISO 22196

The antibacterial effectiveness of neat PP‐Cl and PP‐Cl/N‐BSHC composite films was further evaluated using a method adapted from ISO 22196 to assess contact‐active antibacterial behavior [22]. *S. aureus ATCC 29213* and *E. coli ATCC 25922* were selected as test microorganisms due to their relevance in evaluating surface‐associated bacterial contamination. Film samples were cut into 1 × 1 cm specimens and sterilized prior to testing. Bacterial suspensions were prepared in sterile physiological saline and adjusted to a concentration of 1 × 10^5^ CFU mL^−1^. Each film specimen was placed into a sterile tube, followed by the addition of the bacterial suspension to ensure direct contact between the bacterial cells and the film surface. The samples were incubated at 35°C for 24 h under gentle agitation to maintain uniform exposure. After incubation, the bacterial suspensions were serially diluted and plated onto appropriate agar media. Colony‐forming units (CFUs) were counted after incubation to determine the number of surviving bacteria [20, 23]. The antibacterial activity was expressed as the percentage reduction (*R*%) using Equation (2)

(2) $$R\left(\%\right)=\left(\frac{B-A}{B}\right)\times 100$$

where *B* represents the initial bacterial count and *A* corresponds to the number of viable bacteria recovered after contact with the film.

### 2.7 Instrumentation

FT‐IR spectra of BSHC, N‐BSHC, neat PP‐Cl, and PP‐Cl/N‐BSHC composite films were recorded using a Bruker Tensor II spectrometer equipped with an attenuated total reflectance (ATR) accessory. Spectra were collected over the range of 4000–400 cm^−1^ at room temperature to identify functional groups and to confirm surface modification of the HC. Surface morphology and elemental composition of the samples were examined using a field‐emission SEM (Zeiss EVO LS 10) coupled with an EDX detector. Prior to analysis, the samples were sputter‐coated with a thin gold layer to enhance conductivity. WCA measurements were performed using a contact angle goniometer (NanoLinker, CA‐500 A) via the sessile drop method to evaluate surface wettability. Thermal stability was assessed by TGA using a TG/DTA6200 system (SII NanoTechnology) under a nitrogen atmosphere. Samples were heated from room temperature to 800°C at a constant heating rate of 10°C min^−1^. The corresponding derivative thermogravimetric (DTG) curves were obtained to analyze thermal degradation behavior. DSC measurements were carried out using a Mettler Toledo DSC‐1 Star System under nitrogen flow. Samples were subjected to a heating–cooling–reheating cycle to eliminate thermal history. Initially, the samples were heated to 100°C and held isothermally, cooled to room temperature, and then reheated to 200°C at a rate of 10°C min^−1^. Thermal transition temperatures were determined from the second heating cycle. Mechanical properties of the films were measured at room temperature using a universal testing machine (Zwick/Roell) in accordance with DIN EN ISO 527–1. Mechanical measurements were performed using at least five independent specimens for each formulation, and the reported tensile parameters correspond to average values. Rectangular specimens (10 mm × 50 mm) were tested at a crosshead speed of 5 mm min^−1^. UV–visible absorption measurements for antioxidant activity assays were carried out using an Optizen POP UV/Vis spectrophotometer.

## 3 Results and Discussion

**FIGURE 1:**
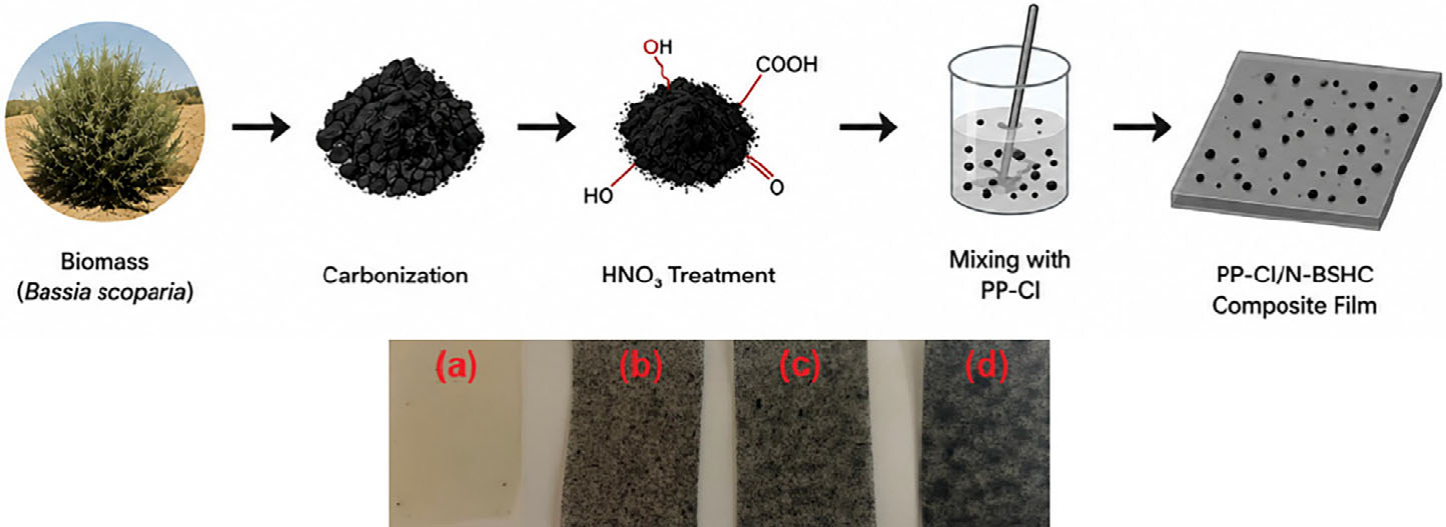
Overall preparation methodology and visual photographs of neat PP‐Cl (a), PP‐Cl/N‐BSHC‐1.0 (b), PP‐Cl/N‐BSHC‐1.5 (c), and PP‐Cl/N‐BSHC‐2.5 (d) prepared with different N‐BSHC loadings.

The development of multifunctional polymer composites has become increasingly important to meet the growing demand for materials that simultaneously exhibit improved surface, thermal, mechanical, and bioactive performance while maintaining processability and sustainability [24]. In this context, incorporating bio‐derived and surface‐functionalized carbon fillers into modified polyolefin matrices represents an effective strategy to overcome the intrinsic limitations of conventional PP‐based systems. The present study was designed to evaluate the synergistic effects of nitric acid‐treated HC derived from *B. scoparia* on the performance of PP‐Cl composites, with particular emphasis on structure–property relationships. Figure 1 presents overall methodology and visual photographs of the solution casted neat PP‐Cl and PP‐Cl/N‐BSHC composite films, providing an initial macroscopic comparison of material appearance prior to detailed spectroscopic and physicochemical analyses.

**FIGURE 2:**
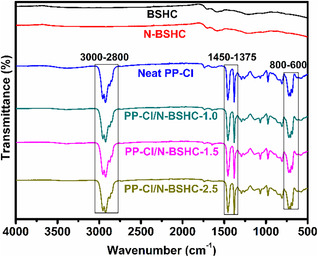
FT‐IR spectra of BSHC, NBSHC, neat PP‐Cl and PP‐Cl/N‐BSHC composite films.

Following the visual inspection, FT‐IR spectroscopy was employed to elucidate the chemical structure of the BSHC, N‐BSHC, neat PP‐Cl, and the corresponding PP‐Cl/N‐BSHC composite films, as shown in Figure 2. The FT‐IR spectrum of pristine BSHC displayed an almost featureless baseline with only weak and broad absorption signals, which was characteristic of highly carbonized materials dominated by condensed aromatic carbon domains. This behavior indicated that most oxygen‐containing functional groups originally present in the biomass precursor were removed during high‐temperature carbonization, resulting in a chemically inert and structurally stable carbon framework [25]. After nitric acid treatment, the FT‐IR spectrum of N‐BSHC remained largely similar to that of pristine BSHC, with no clearly distinguishable new absorption bands attributable to specific oxygen‐ or nitrogen‐containing functional groups. This observation suggests that, despite the oxidative nature of nitric acid, the highly condensed carbon structure limits the extent of surface functionalization detectable by ATR‐FT‐IR. Any oxidation induced by nitric acid treatment is therefore likely confined to a low concentration of surface sites or present at levels below the detection limit of the technique. The FT‐IR spectrum of neat PP‐Cl exhibited characteristic absorption bands associated with the PP backbone, including C—H stretching vibrations in the 3000–2800 cm^−1^ region and bending vibrations near 1450 and 1375 cm^−1^, together with bands related to C—Cl stretching typically observed in the 800–600 cm^−1^ range [26]. In the PP‐Cl/N‐BSHC composite films, the presence of the characteristic bands of PP‐Cl alongside the carbon‐related features confirms the successful incorporation of HC into the polymer matrix. Although no new covalent bond formation is evident from the FT‐IR spectra, subtle changes in band intensity and profile indicate physical interactions and improved interfacial compatibility between PP‐Cl and N‐BSHC, which contribute to the enhanced surface, thermal, and bioactive properties discussed in the subsequent sections.

**FIGURE 3:**
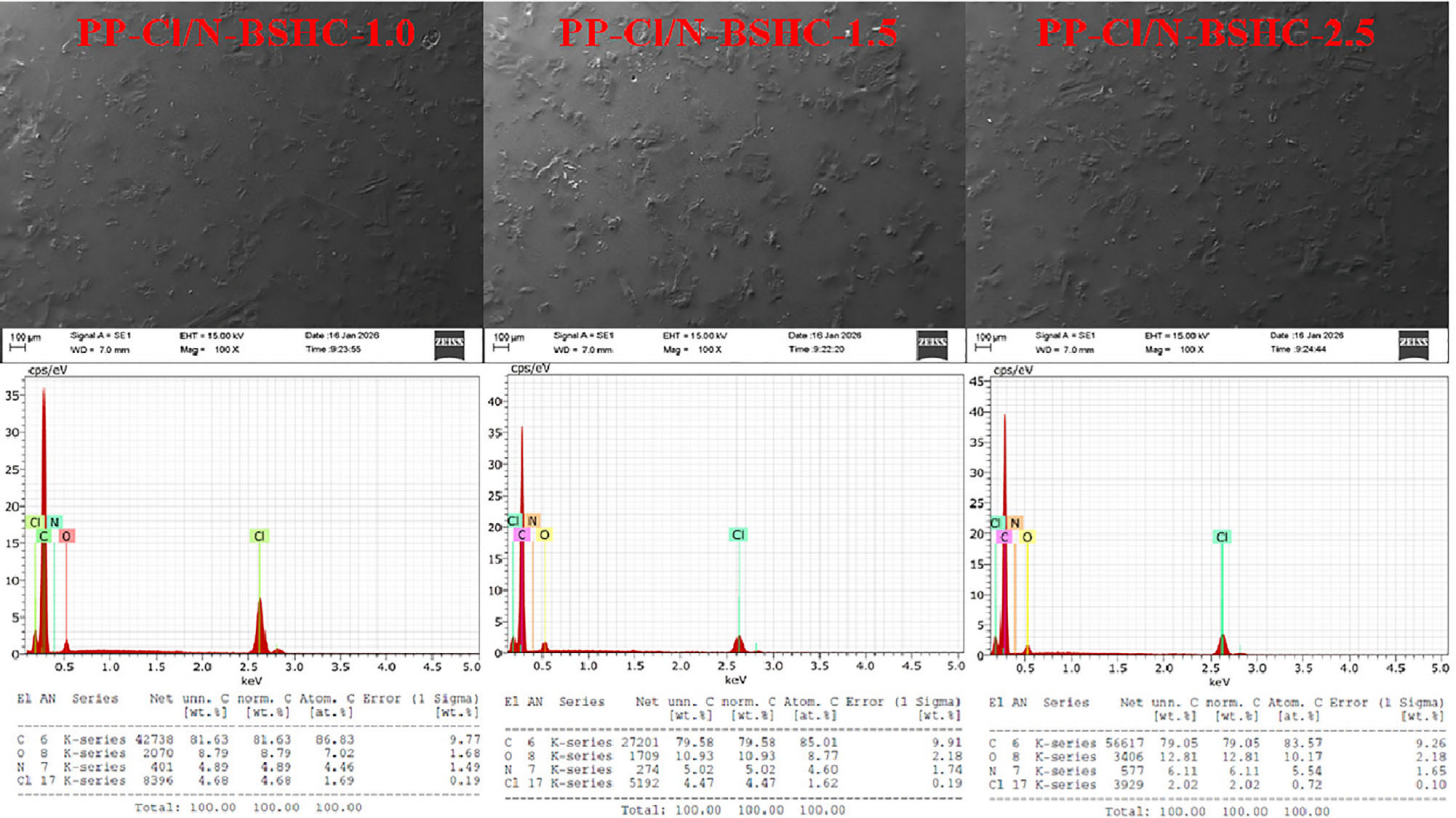
SEM micrographs, EDX spectra, and corresponding elemental atomic percentage analysis of PP‐Cl/N‐BSHC composite films prepared with different N‐BSHC loadings. Preliminary observations for untreated BSHC‐filled composites showed less homogeneous filler distribution compared with N‐BSHC‐containing systems.

The surface morphology and elemental composition of the PP‐Cl/N‐BSHC composite films were investigated by SEM coupled with EDX analysis, and the results are presented in Figure 3. The SEM micrographs revealed a continuous polymeric matrix containing embedded HC particles, confirming the successful incorporation of N‐BSHC into the PP‐Cl matrix. The filler particles were distributed throughout the surface without the presence of large agglomerates, indicating effective dispersion achieved through nitric acid treatment of the HC and the use of a dispersing agent during composite preparation. With increasing N‐BSHC content, a gradual increase in surface roughness was observed, which was consistent with the higher fraction of rigid carbonaceous domains exposed at the film surface. EDX spectra of the composite films showed carbon (C), chlorine (Cl), oxygen (O), and nitrogen (N) as the principal detected elements. Carbon originates from both the PP‐Cl matrix and the HC filler, while chlorine confirms the presence of the chlorinated PP backbone. The detection of oxygen and nitrogen was attributed to the nitric acid‐treated HC, indicating the introduction of oxygen‐ and nitrogen‐containing surface functionalities during the oxidative treatment. Quantitative atomic percentage values obtained from the EDX analysis, presented in the table below the spectra, further substantiate these observations. With increasing N‐BSHC content, the atomic percentages of carbon, oxygen, and nitrogen increased progressively, accompanied by a corresponding decrease in chlorine content. This compositional evolution reflects the growing contribution of the nitric acid‐treated HC phase within the PP‐Cl matrix. The presence and gradual increase of nitrogen, although relatively low in concentration, provide additional evidence of surface modification induced by nitric acid treatment and may contribute to enhanced interfacial interactions. Preliminary experiments performed using untreated BSHC‐filled PP‐Cl composites revealed comparatively weaker dispersion behavior and lower improvements in surface and bioactive properties than those observed for nitric acid‐treated N‐BSHC systems. These observations suggest that nitric acid oxidation plays a critical role in improving matrix‐filler compatibility through the introduction of surface polar functionalities.

**FIGURE 4:**
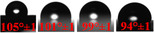
WCA images and average values of neat PP‐Cl and PP‐Cl/N‐BSHC composite films with increasing N‐BSHC content from left to right.

The surface wettability of neat PP‐Cl and PP‐Cl/N‐BSHC composite films was evaluated by WCA measurements, and the results were presented in Figure 4. Compared with preliminary untreated BSHC systems, N‐BSHC‐containing composites exhibited a more pronounced reduction in water contact angle, indicating improved surface polarity and wettability after nitric acid treatment. Neat PP‐Cl exhibited a relatively high WCA of 105° ± 1°, reflecting the inherently hydrophobic nature of the PP backbone despite chlorination. Upon incorporation of nitric acid‐treated HC, a gradual decrease in WCA was observed with increasing N‐BSHC content, with values of 101° ± 1°, 99° ± 1°, and 94° ± 1° for PP‐Cl/N‐BSHC‐1.0, PP‐Cl/N‐BSHC‐1.5, and PP‐Cl/N‐BSHC‐2.5, respectively. The progressive reduction in WCA indicates enhanced surface wettability of the composite films, which can be attributed to the combined effects of surface chemistry and morphology. As demonstrated by SEM‐EDX analysis, the incorporation of N‐BSHC introduces oxygen‐ and nitrogen‐containing surface functionalities that increase the polar character of the film surface [27, 28]. In addition, the increased surface roughness observed at higher N‐BSHC loadings contributes to greater exposure of these polar sites, facilitating stronger interactions between the surface and water droplets.

**FIGURE 5:**
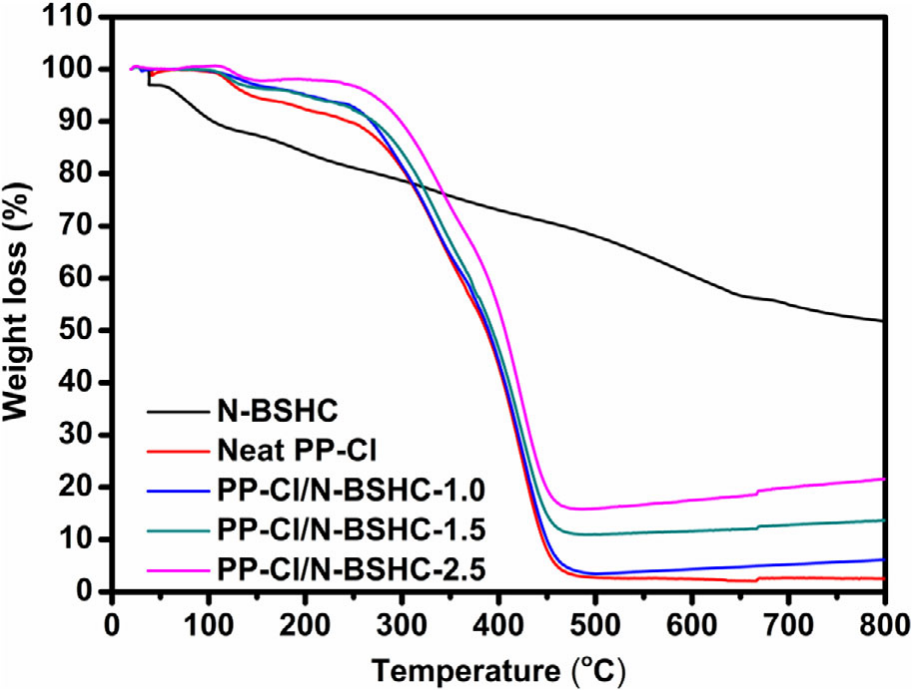
TGA curves of N‐BSHC, neat PP‐Cl, and PP‐Cl/N‐BSHC composite films recorded under nitrogen atmosphere at a heating rate of 10°C min^−1^.

TGA was conducted under nitrogen to investigate the thermal degradation behavior of N‐BSHC, neat PP‐Cl, and the composite films (Figure 5). N‐BSHC exhibited high intrinsic thermal stability, showing a gradual single‐step mass loss and retaining 51.26% of its initial mass at 800°C, which is characteristic of highly condensed HC structures. All samples displayed a minor initial weight loss of ≈3%–5% in the 100°C–150°C region, attributed to the removal of physically adsorbed moisture, residual solvent, and labile surface functionalities rather than polymer degradation. Neat PP‐Cl exhibited a characteristic two‐stage degradation behavior associated with dehydrochlorination followed by backbone scission [29]. The maximum degradation temperatures observed from the main mass‐loss regions of the TGA curve were 338°C and 421°C. Upon incorporation of N‐BSHC, the composite films showed a progressive shift of these maximum degradation temperatures toward higher values, reaching 340°C/423°C for PP‐Cl/N‐BSHC‐1.0, 343°C/425°C for PP‐Cl/N‐BSHC‐1.5°C, and 346°C/428°C for PP‐Cl/N‐BSHC‐2.5. In addition, the residual mass increased markedly from 2.54% for neat PP‐Cl to 6.23%, 13.78%, and 21.97% with increasing filler content. The systematic increase in degradation temperatures and char residue indicates that the thermally stable HC phase acts as a protective barrier, limiting heat transfer and slowing the evolution of volatile decomposition products, thereby enhancing the overall thermal resistance of the PP‐Cl matrix in a filler‐content‐dependent manner [30, 31]. The gradual mass loss observed for N‐BSHC does not indicate incomplete carbonization but is mainly associated with the decomposition of residual surface functionalities and disordered carbon domains generated during nitric acid oxidation. Biomass‐derived HCs commonly exhibit such broad thermal behavior due to the presence of oxygen‐ and nitrogen‐containing groups on the carbon surface [32, 33]. The high residual mass retained at 800°C confirms the formation of a thermally stable HC structure.

**FIGURE 6:**
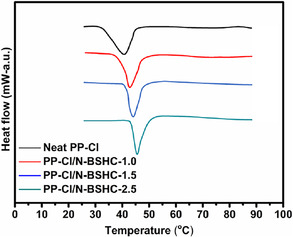
DSC thermograms of neat PP‐Cl and PP‐Cl/N‐BSHC composite films recorded under nitrogen atmosphere during the second heating cycle.

The thermal transition behavior of neat PP‐Cl and PP‐Cl/N‐BSHC composite films was investigated by DSC, and the corresponding thermograms are presented in Figure 6. Distinct melting (*T*_m_) and crystallization (*T*_c_) transitions were not clearly observed in the DSC thermograms due to the partially amorphous nature of chlorinated PP. Therefore, reliable crystallinity calculations could not be performed, and the discussion was mainly focused on the glass transition behavior (*T*_g_) of the composite systems. The *T*_g_ of neat PP‐Cl was determined to be 40.1°C, reflecting the inherent chain mobility of the PP‐Cl matrix. Upon incorporation of nitric acid‐treated HC, a systematic increase in *T*_g_ was observed, with values of 42.5°C, 44.1°C, and 45.3°C for PP‐Cl/N‐BSHC‐1.0, PP‐Cl/N‐BSHC‐1.5, and PP‐Cl/N‐BSHC‐2.5, respectively. The progressive increase in *T*_g_ indicates restricted segmental motion of the PP‐Cl chains in the presence of N‐BSHC [34, 35]. This behavior can be attributed to enhanced interfacial interactions between the polymer matrix and the HC filler, as well as the physical confinement effect imposed by the rigid carbonaceous domains [36]. Although a direct quantitative crystallinity analysis could not be reliably performed due to the absence of well‐defined melting and crystallization transitions in the DSC thermograms of PP‐Cl, the results suggest that N‐BSHC incorporation promotes a more constrained polymer structure rather than significantly increasing crystalline ordering. The partially amorphous nature of chlorinated PP and the structural irregularities introduced by chlorination likely limit the development of highly ordered crystalline domains within the composite system.

**FIGURE 7:**
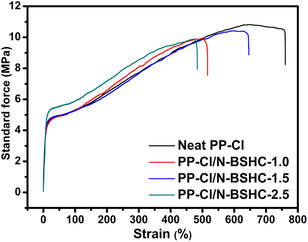
Representative tensile standard force‐strain curves of neat PP‐Cl and PP‐Cl/N‐BSHC composite films with different N‐BSHC loadings.

The tensile behavior of neat PP‐Cl and PP‐Cl/N‐BSHC composite films was evaluated by uniaxial tensile testing, and the corresponding curves are presented in Figure 7, with the mechanical parameters summarized in Table 1. Neat PP‐Cl exhibited a typical ductile deformation behavior characterized by a relatively low elastic modulus, moderate tensile strength, and high elongation at break. This response reflects the flexible nature of the PP‐Cl matrix and serves as a reference for assessing the effect of HC incorporation. Upon addition of nitric acid‐treated HC, a clear stiffening effect was observed [37]. The elastic modulus (*ε*_t_) increased from 48.56 MPa for neat PP‐Cl to 61.22, 73.61, and 70.62 MPa for PP‐Cl/N‐BSHC‐1.0, PP‐Cl/N‐BSHC‐1.5, and PP‐Cl/N‐BSHC‐2.5, respectively. The stiffening effect was attributed to the rigid carbonaceous structure of N‐BSHC and effective stress transfer across the matrix–filler interface, as supported by the homogeneous dispersion observed in SEM analysis [38]. The tensile strength values showed a more complex trend. While the maximum stress (*σ*_m_) remained comparable to neat PP‐Cl, slight variations were observed depending on filler loading. The PP‐Cl/N‐BSHC‐1.5 sample exhibited the highest tensile strength (10.42 MPa), suggesting an optimal balance between filler reinforcement and matrix continuity. At higher filler content (2.5 wt%), a slight reduction in tensile strength was observed, which may be related to local stress concentration effects induced by increased filler loading. In contrast to the increase in stiffness, the elongation at break (*ε*_b_) and toughness decrease progressively with N‐BSHC incorporation. Neat PP‐Cl showed a high elongation at break of 760.74%, whereas the composite films exhibited reduced values of 516.09%, 645.94%, and 484.04% for increasing N‐BSHC content. This trend was clearly visible in Figure 7, where the composite films displayed shorter deformation regions prior to fracture. Similarly, the fracture energy (W(break)) decreased from 4876.35 N.mm for neat PP‐Cl to 1888.05 N.mm for PP‐Cl/N‐BSHC‐2.5, indicating reduced energy absorption capability due to restricted polymer chain mobility.

**TABLE 1:** Tensile properties of neat PP‐Cl and PP‐Cl/N‐BSHC composite films with different N‐BSHC loadings, including elastic modulus (*ε*_t_), maximum tensile stress (*σ*_m_), strain at maximum stress (*ε*_m_), tensile stress at break (*σ*_b_), elongation at break (*ε*_b_), and fracture energy (W(break)).

Sample	*ε*_t_, MPa	*σ*_m_, MPa	*ε*_m_, %	*σ*_b_, MPa	*ε*_b_, %	W(break), N.mm
PP‐Cl	48.56	10.82	647.61	8.24	760.74	4876.35
PP‐Cl/N‐BSHC‐1.0	61.22	9.95	501.34	7.55	516.09	3292.58
PP‐Cl/N‐BSHC‐1.5	73.61	10.42	596.04	8.87	645.94	2672.45
PP‐Cl/N‐BSHC‐2.5	70.62	9.88	472.18	7.95	484.04	1888.05

**FIGURE 8:**
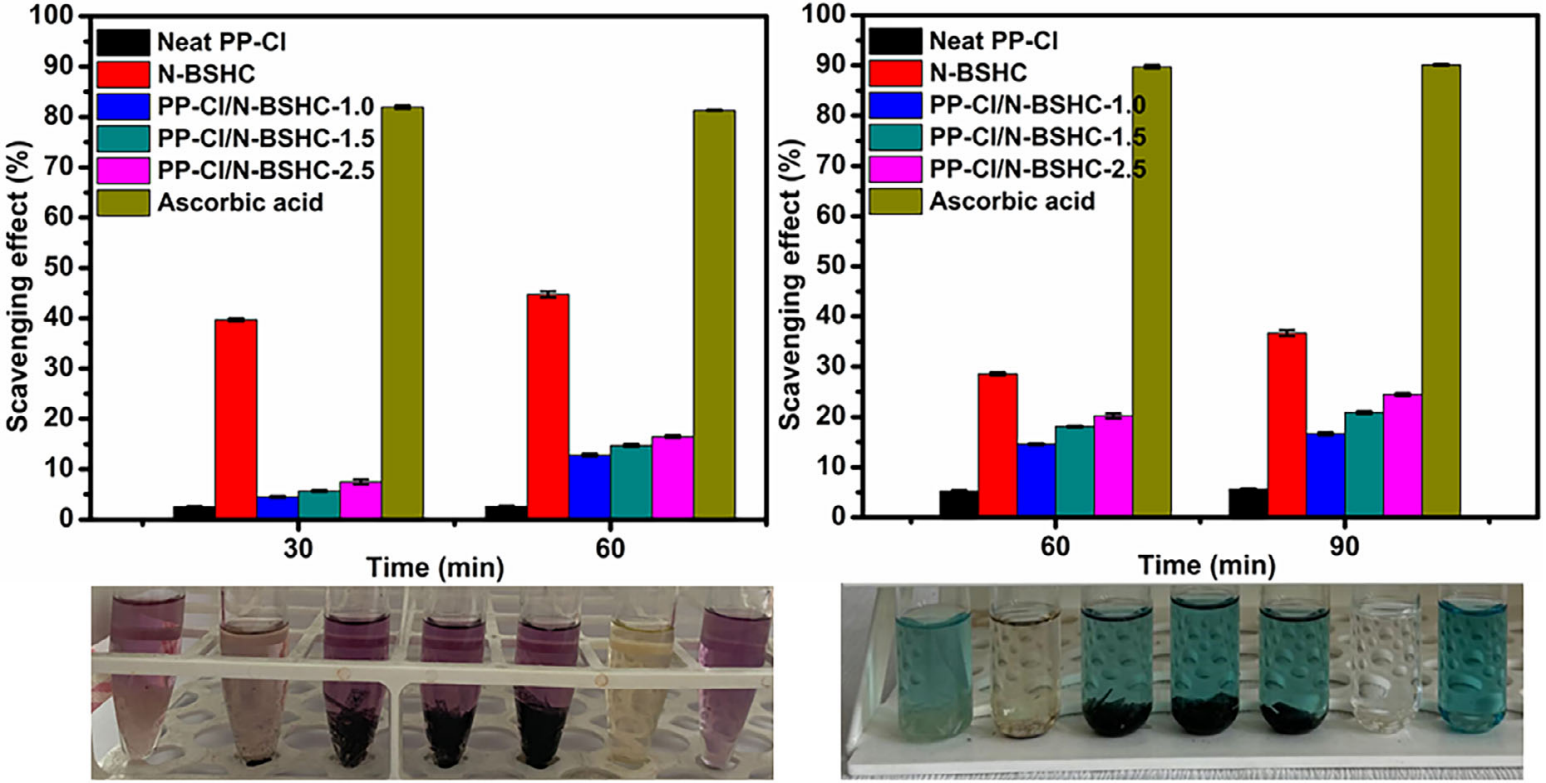
DPPH and ABTS radical scavenging activities of N‐BSHC, neat PP‐Cl, and PP‐Cl/N‐BSHC composite films.

The antioxidant activity of N‐BSHC, neat PP‐Cl, and PP‐Cl/N‐BSHC composite films was evaluated using DPPH and ABTS radical scavenging assays (Figure 8). Neat PP‐Cl exhibited negligible radical scavenging activity, with DPPH and ABTS inhibition values of 2.52% and 2.62% after 30 and 60 min and 5.23% and 5.58% after 60 and 90 min respectively, which was consistent with the chemically inert nature of the PP backbone. In contrast, N‐BSHC demonstrated measurable antioxidant activity, with DPPH and ABTS radical scavenging efficiencies of 39.68% and 44.77% and 28.57% and 36.69%, respectively, after the same periods. This behavior was attributed to the presence of surface‐active sites and residual functional groups on the carbon surface, which are capable of interacting with and neutralizing free radicals. The PP‐Cl/N‐BSHC composite films showed a clear enhancement in antioxidant performance compared to neat PP‐Cl. The radical scavenging activity increased progressively with increasing N‐BSHC content. Specifically, the DPPH inhibition values reach 4.52%–12.69%, 5.65%–14.71%, and 7.48%–16.46% for PP‐Cl/N‐BSHC‐1.0, PP‐Cl/N‐BSHC‐1.5, and PP‐Cl/N‐BSHC‐2.5, respectively. A similar trend was observed in the ABTS assay, with inhibition values of 14.61%–16.65%, 18.10%–20.89%, and 20.20%–24.46% for the corresponding composite films. The gradual improvement in antioxidant activity with increasing filler content confirmed that nitric acid‐treated HC acts as an effective antioxidant‐active component within the PP‐Cl matrix [39, 40, 41].

The antibacterial performance of N‐BSHC and PP‐Cl/N‐BSHC composite films was evaluated using broth microdilution assays and a contact‐based antibacterial test adapted from ISO 22196. The results of the MIC tests against *S. aureus* ATCC 29213 and *E. coli* ATCC 25922 were summarized in Table 2, while the surface‐associated antibacterial activity of the composite films was presented in Table 3. As shown in Table 2, N‐BSHC exhibited antibacterial activity against both *S. aureus* and *E. coli*, with MIC values of 128 µg mL^−1^ for each strain. Although these values were higher than those of conventional antibiotics used as reference controls, they clearly demonstrated the intrinsic antibacterial potential of the nitric acid‐treated HC. The comparable MIC values observed for Gram‐positive and Gram‐negative bacteria indicated that the antibacterial activity of N‐BSHC was not strongly strain‐dependent and is likely associated with surface‐mediated interactions rather than specific biochemical targets. The antibacterial performance of the composite films was further evaluated under direct contact conditions using the ISO 22196‐based method (Table 3). Neat PP‐Cl showed only limited antibacterial activity, with reduction rates of 19% and 18% against *S. aureus* and *E. coli*, respectively, confirming the absence of inherent antimicrobial functionality in the polymer matrix. In contrast, the PP‐Cl/N‐BSHC composite films exhibited a pronounced and filler‐content‐dependent antibacterial effect. For *S. aureus*, the bacterial reduction increased from 41% for PP‐Cl/N‐BSHC‐1.0 to 60% for PP‐Cl/N‐BSHC‐1.5, reaching 96% for PP‐Cl/N‐BSHC‐2.5. A similar trend was observed for *E. coli*, with reduction rates of 42%, 63%, and 97% for the corresponding composite films. The progressive increase in antibacterial efficiency with increasing N‐BSHC content highlighted the critical role of nitric acid‐treated HC in imparting antibacterial functionality to the PP‐Cl matrix. This behavior can be attributed to the combined effects of increased surface roughness, the presence of oxygen‐ and nitrogen‐containing surface functionalities, and enhanced interfacial exposure of the carbon filler at the film surface, as evidenced by SEM‐EDX and WCA analyses [42, 43]. The high antibacterial reduction achieved at 2.5 wt% N‐BSHC demonstrated that effective contact‐active antibacterial surfaces can be obtained without the use of conventional biocidal additives or metal‐based agents.

**TABLE 2:** MIC values of N‐BSHC and standard antibiotics against *S. aureus* ATCC 29213 and *E. coli* ATCC 25922.

Samples	*S. aureus* ATCC 29213	*E. coli* ATCC 25922
N‐BSHC	128	128
Ampicillin	0.5	8
Vancomycin	0.5	—
Cefotaxime	1	0.250
Ciprofloxacin	0.5	0.016
Gentamicin	0.25	0.5
Imipenem	—	0.125

**TABLE 3:** Colony count changes and antibacterial reduction rates (R%) of neat PP‐Cl and PP‐Cl/N‐BSHC composite films against *S. aureus* ATCC 29213 and *E. coli* ATCC 25922 determined using an ISO 22196‐based contact test.

Microorganism	Initial inoculum	Neat PP‐Cl, CFU/mL	R, %	PP‐Cl/N‐BSHC‐1.0, CFU/mL	R, %	PP‐Cl/N‐BSHC‐1.5, CFU/mL	R, %	PP‐Cl/N‐BSHC‐2.5, CFU/mL	R, %
*S. aureus* ATCC 29213	1.0 × 10^5^	8.1 × 104	19	5.9 × 10^4^	41	4.0 × 10^4^	60	4.1 × 10^3^	96
*E. coli ATCC 25922*	1.0 × 10^5^	8.2 × 104	18	5.8 × 10^4^	42	3.7 × 10^4^	63	3.5 × 10^3^	97

## 4 Conclusions

In this study, bio‐derived HC obtained from *B. scoparia* and subsequently treated with nitric acid was successfully incorporated into PP‐Cl to fabricate multifunctional composite films. The results demonstrated that the nitric acid‐treated HC (N‐BSHC) acted as an effective reinforcement and functional additive, enabling simultaneous improvements in surface, thermal, mechanical, antioxidant, and antibacterial properties of the PP‐Cl matrix. SEM‐EDX analysis confirmed homogeneous dispersion of N‐BSHC within the PP‐Cl matrix and revealed the presence of oxygen‐ and nitrogen‐containing surface functionalities, which contributed to enhanced interfacial compatibility. Surface wettability measurements showed a systematic decrease in water contact angle from 105° for neat PP‐Cl to 94° for the composite containing 2.5 wt% N‐BSHC, indicating tunable surface hydrophilicity. Thermal analyses revealed improved thermal stability and restricted polymer chain mobility in the composite films, as evidenced by increased degradation resistance and a progressive increase in glass transition temperature. Mechanical testing demonstrated a significant increase in elastic modulus with increasing N‐BSHC content, accompanied by a moderate reduction in ductility, confirming the reinforcing role of the rigid carbonaceous filler. Notably, the composite containing 1.5 wt% N‐BSHC exhibited a balanced combination of stiffness, strength, and elongation. Furthermore, antioxidant assays confirmed that the incorporation of N‐BSHC imparted radical scavenging activity to the otherwise inert PP‐Cl matrix, while antibacterial evaluations revealed filler‐content‐dependent antibacterial performance against both *S. aureus* and *E. coli*, achieving up to 96%–97% bacterial reduction. Overall, this work demonstrates that nitric acid‐treated HC derived from *B. scoparia* is sustainable and metal‐free multifunctional filler capable of significantly enhancing the performance of PP‐Cl‐based composites. The developed composite films show strong potential for advanced coating and packaging applications where improved thermal resistance, surface functionality, and bioactive performance are required such as multifunctional protective film and active packaging applications. The use of biomass‐derived precursor materials, low filler loading, and relatively simple processing methods suggests potential scalability and industrial applicability of the developed composite system. However, detailed techno‐economic analysis and large‐scale production evaluation were beyond the scope of the present study and should be investigated in future work. Despite the promising sustainability aspects associated with the use of biomass‐derived HC, it should be noted that a comprehensive environmental evaluation of the developed composite system requires a detailed LCA. In particular, the energy demand of the carbonization process, chemical consumption during nitric acid oxidation, solvent usage, and potential emissions associated with large‐scale production should be quantitatively assessed to determine the overall environmental footprint of the material. Therefore, future studies should include cradle‐to‐grave or cradle‐to‐gate LCA analyses to evaluate the environmental benefits and potential trade‐offs of the proposed biomass‐derived multifunctional composite system in comparison with conventional petroleum‐based or mineral‐filled polymer composites.

## Author Contributions

**Gokhan Acik**: visualization, methodology, formal analysis, data curation, investigation, writing – original draft, writing – review & editing. **Esra Kulaksiz Alayont**: investigation, formal analysis, data curation. **Burcu Acik**: writing – review & editing, investigation, formal analysis, data curation. **Gulcan Kuyucuklu Kazan**: investigation, formal analysis, data curation. **Derya Altintas**: investigation, formal analysis, data curation. **Hakan Kolancilar**: formal analysis. **Yuksel Bayrak**: investigation, writing – review & editing.

## Funding

This research did not receive any specific grant from funding agencies in the public, commercial, or not‐for‐profit sectors.

## Conflicts of Interest

The authors declare no conflicts of interest.

## Data Availability

Data available on request from the authors.
